# Symptoms of Anxiety and Depression Are Correlates of Angina Pectoris by Recent History and an Ischemia-Positive Treadmill Test in Patients with Documented Coronary Artery Disease in the Pimi Study

**DOI:** 10.1155/2011/134040

**Published:** 2011-11-17

**Authors:** Mark W. Ketterer, Nadine S. Bekkouche, A. David Goldberg, Robert P. McMahon, David S. Krantz

**Affiliations:** ^1^Consultation/Liaison Psychiatry, Henry Ford Hospital and Wayne State University, Detroit, MI 48202, USA; ^2^Department of Medical & Clinical Psychology, Uniformed Services University of the Health Sciences, Bethesda, MD 20814, USA; ^3^Department of Cardiology, University of Michigan, Ann Arbor, MI 48109, USA; ^4^Department of Psychiatry, University of Maryland, Catonsville, MD 21228, USA

## Abstract

*Objective*. We tested the association of specific psychological characteristics in patients having stable coronary disease with the reporting of anginal symptoms during daily activities, and positive exercise testing. *Methods*. One hundred and ninety-six patients with documented CAD enrolled in the Psychophysiological Investigations of Myocardial Ischemia (PIMI) Study completed an anginal history questionnaire and a battery of psychometric tests. They also underwent standardized exercise treadmill tests. *Results*. Patients with a recent history of angina were more likely to be female, and had higher Beck Depression (*P* = .002), State Anxiety (*P* = .001), Trait Anxiety (*P* = .03), Harm Avoidance (*P* = .04) and Muscle Tension (*P* = .004) scores than patients who had no recent history of angina. Along with several treadmill variables indicating more severe disease state and reduced exercise tolerance, patients who developed angina on a positive treadmill test also displayed higher scores on the Beck Depression Inventory (*P* = .003) and State Anxiety (*P* = .004) scales. *Conclusions*. Several psychological characteristics, and most notably anxiety and depression, are strong correlates of recent angina and angina in the presence of ischemia provoked by treadmill testing.

## 1. Introduction

Since the term “angina pectoris” was first coined and recognized as a prodrome to sudden cardiac death [1, 2], the causes of chest pain/discomfort have been a topic of investigation and intervention. It is now known that although myocardial ischemia sometimes causes anginal symptoms, the majority of ischemic episodes are silent. Conversely, most episodes of angina-like chest pain in patients known to have coronary artery disease are unaccompanied by measurable ischemia [3]. The factors that determine whether or not angina occurs during ischemia are controversial. Recent studies have identified a variety of physiological correlates, including the type of triggering activity [3], the severity of ischemia or ventricular dysfunction [4], adenosine release [5], endogenous opiate response [6–8], and pain threshold [9].

 A variety of psychological characteristics have also been found to be associated with reporting, or denying, anginal symptoms [10–24], or with clinical manifestations of disturbed ischemic pain perception/coping such as treatment seeking delay during acute myocardial infarction [25], silent myocardial infarction [26, 27], or unheralded sudden cardiac death [28]. It is difficult to compare these studies because they are not entirely consistent with respect to the psychological variables that were included, or the methods and measures used. A principal aim of the Psychophysiological Investigations of Myocardial Ischemia (PIMI) study was to concurrently assess, in a large and well-defined sample of patients with known coronary disease, many of the psychological factors that may be associated with the reporting/denying of anginal symptoms during daily life or on exercise provocation.

On the basis of the earlier studies, we predicted that patients with coronary artery disease who report angina during daily activities, and on a positive exercise treadmill test (ETT) would also report more physical symptoms, and have higher average levels of psychological characteristics or symptoms of such states as anxiety and depression, than patients who develop silent ischemic on the same ETT.

## 2. Methods

Details on the overall protocol of the PIMI Study have been published elsewhere [29–34]. The present study utilizes clinical/demographic data gathered during the recruitment phase of PIMI, results of the baseline qualifying treadmill exam (which was required to be positive for ischemia to achieve eligibility), and psychometric data gathered at a later visit. 

### 2.1. Study Patients

A total of 196 patients (26 females) with stable coronary artery disease (confirmed by positive catheterization and/or past myocardial infarction) who developed myocardial ischemia on a standardized, symptom-limited exercise treadmill test were enrolled in the PIMI Study. All patients were withdrawn from cardiovascular and psychoactive medications three days prior to the qualifying treadmill exam.

### 2.2. Medical Assessments

Medical history data were obtained from a standardized medical history form. Presence of angina within the last three months was determined using the Rose Questionnaire [35], which was collected as a subscale of the Anginal Syndrome Questionnaire [36]. A screening examination to exclude patients with cognitive impairment or peripheral neuropathy was used for entry to the study.

### 2.3. Exercise Testing

All patients underwent a standardized, symptom-limited exercise treadmill test using the Asymptomatic Cardiac Ischemia Pilot (ACIP) study ETT protocol [37]. The electrocardiographic criteria for myocardial ischemia were met if either: (a) J point depression ≥0.1 mm, and horizontal or >1 mm/second downsloping ST segment, and ST-80 depression ≥0.15 mm, or (b) ST-80 depression ≥0.15 mm and T segment sloping upward >1 mm/second were present during the treadmill test.

### 2.4. Psychometric Battery

Subjects were also scheduled to return for psychological testing within three months after completing the ETT. The psychometric battery consisted of seven questionnaires administered by study personnel; the Beck Depression Inventory (BDI)—a 21-item measure of the severity of depressive symptoms [38]; the State-Trait Anxiety Inventory (STAI)—a measure of acute and chronic anxiety [39]; a modified version of the Autonomic Perception Questionnaire (MAPQ) which included subscales on Autonomic Perception and Muscle Tension [40]; the Toronto Alexithymia Scale (TAS)—a 26-item measure of awareness of the external environment to the exclusion of self-awareness [41]; the 24-item Anger Expression Scale (AX) which includes subscales for Anger Out (AXOUT) to assess overtly expressed anger, Anger In (AXIN) to assess unexpressed anger, and Anger Control (AXCON) to assess efforts to suppress the overt expression of anger [42]; the Cook-Medley Hostility Scale (CMS)—a 50-item measure of hostile attitudes [43]; the Tridimensional Personality Questionnaire (TPQ) which includes the 35-item Harm Avoidance Scale (HA) to assess the tendency to avoid stimuli perceived to be potentially harmful or painful, the 40-item Novelty Seeking Scale (NA) to assess the tendency to respond strongly to novel stimuli and to cues for relief from aversive stimulation, the 24-item Reward Dependence Scale (RD) to assess the tendency to respond strongly to social approval and other sources of positive reinforcement [44].

### 2.5. Statistical Analysis

The normal scores rank test was used instead of the two-sample *t*-test to compare continuously distributed variables in order to protect against the effects of outliers and nonnormal distributions [45, 46]. Pearson's chi-square test was used to test for association between discrete variables. In order to conduct a Stepwise Cox Regression of angina on the treadmill test using risk factors known to covary with angina (Sex and History of Hypertension-[22]) as well as two indices of disease severity (Time to Initial ST Segment Depression and Rate-Pressure Product at Initial ST segment depression) and State Anxiety and Depression, patients without angina were scored as having Angina onset at one minute past the maximum number of minutes any patient stayed on the test. A Stepwise Logistic Regression using the same control variables was used to conduct the same analysis for angina by history.

## 3. Results

### 3.1. History of Angina

Seventy patients had a history of angina by Rose criteria during the three months prior to entering the study, while 126 had no such history.  Table 1 displays comparisons for the demographic and medical characteristics between patients who reported a history of anginal symptoms during the three months prior to the study, and those who were asymptomatic during this period. Patients with a recent history of angina were more likely to be female but were otherwise not different on clinical/demographic variables. The patients with recent angina did, however, tend to score higher on the Beck Depression Inventory, the State Anxiety Scale, the Trait Anxiety Scale, and the Harm Avoidance and Muscle Tension subscales. See Table 1. In the Stepwise Logistic Regression (chi-square = 15.1, df = 2, *P* = 0.001), only time to ST segment depression (*B* = −.107) and State Anxiety (*B* = .055) survived as unique predictors of angina by history.

### 3.2. Exercise Treadmill Test

Ninety patients developed angina during the qualifying treadmill test, while 106 did not. As shown in Table 2, there were no differences on clinical/demographic characteristics between patients experiencing angina on the ischemia-positive ETT and those who did not. However, patients with angina had evidence of more severe disease (shorter Time to 1 mm ST Depression and lower Rate-Pressure Product at ST Onset) and diminished exercise tolerance (less Total ETT Duration, Time to Ends of Test from ST Onset, and lower Rate-Pressure Product at Peak Exercise). Patients who reported angina on the positive ETT displayed higher average scores on the Beck Depression Inventory and the State Anxiety Scale. In the Stepwise Cox Regression (chi-square = 17.2, df = 2, *P* < 0.001), only time to ST segment depression (*B* = −.165) and Depression (*B* = 0.051) retained unique variance.

## 4. Discussion

Our results have demonstrated that patients with angina have a higher frequency of depressive/anxious symptoms. The relationship between angina pectoris in daily life and psychological characteristics has become a topic of some interest in recent years. There have also been several studies examining psychological characteristics in coronary patients with, and without, angina in response to treadmill exercise testing. These studies, while only correlational in nature, are important for several reasons. First they may provide some insight into clinical situations in which angina as a warning sign seems to fail, such as excessive treatment seeking delay during acute myocardial infarction [25], diagnosis of acute myocardial infarction [47], silent myocardial infarction [26, 27], and unheralded sudden cardiac death [28]. Second, they may also carry some implications for controlling angina if the psychological correlates are found to be the cause, rather than the result, of the symptoms. Indeed, several studies already indicate that treatment of emotional distress with cognitive-behavioral therapy reduces angina symptoms [48–57]. Efficacy of psychopharmacological interventions for anxiety/depression in reducing angina in this population is not yet demonstrated, but such studies are urgently needed. 

The present study is unique in that it permits comparisons, within a single sample, of patients both with and without a recent history of angina, and those who are asymptomatic or not on an ischemia-positive treadmill test. The size of the present sample is large in comparison to other studies, the psychometric assessment is broad and comprehensive, and the well-defined baseline characteristics of the sample are also unique. The study may be limited, however, by the fact that the psychometric tests were not administered on the same day as the treadmill exam. This may have weakened the correlations between psychometric tests and angina, since the patient's mood state may have changed in the intervening interval.

The State Anxiety Scale and Beck Depression Inventory were strongly associated with Angina both by recent history, and on a positive treadmill test. Trait Anxiety, Harm Avoidance, and Muscle Tension were also found to be associated with angina by recent history. Alexithymia was unrelated to the presence or absence of angina. The adverse impact of alexithymia on treatment-seeking behavior [25] may not be directly mediated by symptom burden. Anger was also unrelated to the presence or absence of angina. 

This may appear paradoxical to those who assume that ischemia is the principle or only cause of angina in the CAD patient, since even artificial, contrived, and brief anger-evoking situations are known to cause ischemia in about half of patients with documented CAD [58]. We interpret this as indicating that the psychological causes of ischemia and angina may be distinct.

Several studies have now reported that patients who experience angina are more aware of physical symptoms in general than are those with silent ischemia [59]. Our results raise the question of whether somatic hypersensitivity, a well-known and common manifestation of depression and anxiety [60], might not determine whether patients with coronary artery disease experience or report angina. A number of chronic pain conditions are known to lower pain thresholds [61, 62]. This suggests that treatment of anxiety and depression might help to control anginal symptoms (and thus treatment costs) in at least some patients. Healthcare costs are known to be elevated in emotionally distressed CAD patients [63–69]. While these increased costs may be due to more rapid disease progression in some patients [70–73], it also seems likely that greater symptom burden, including other symptoms thought to be caused by ischemic heart disease [22, 74] would increase treatment-seeking for patients, and testing/admission rates for physicians. 

In several studies, treatment of distress has been found to reduce utilization and costs [21, 75–77]. Aggressive, angina-targeted clinical trials that also evaluate impact on utilization, and trials using modern psychopharmacological agents, are needed.

## 5. Conclusions

Patients with known coronary artery disease who report experiencing angina, either by recent history or an ischemia-positive exercise treadmill test, are more likely to report anxious and depressive symptoms. Heightened awareness of physical symptoms, and possibly higher levels of anxiety and depression, differentiate between patients who report angina and those who do not. This raises the question of whether treatment of anxiety and depression may help to diminish angina.

## Figures and Tables

**Table 1:** Demographic, medical, and psychological characteristics of patients with versus without a recent (past three months) history of anginal symptoms.

	Recent anginal symptoms		
Variable	Present	Absent	*P*
	(*N* = 70)	(*N* = 126)	
	%	%	
Male	80%	90.5%	.04
Caucasian	82.9%	89.7%	.17
History of Diabetes	15.7%	14.3%	.79
History of Myocardial Infarction	41.4%	42.9%	.85
History of Hypertension	54.3%	43.7%	.15
History of PTCA	35.7%	34.9%	.91

Variable	M (SD)	M (SD)	*P*

Age	61.4 (8.7)	63.1 (8.2)	.17
Beck Depression Inventory	7.3 (5.7)	5.2 (5.7)	.002
State Anxiety	34.5 (9.8)	29.8 (9.5)	.001
Trait Anxiety	34.3 (8.7)	31.6 (8.7)	.03
Novelty Seeking	16.5 (6.2)	16.5 (5.6)	.97
Harm Avoidance:	13.0 (6.2)	11.1 (7.3)	.04
Reward Dependence:	15.8 (4.4)	15.8 (3.7)	.98
Cook-Medley Hostility Scale	19.9 (8.0)	20.1 (8.7)	.97
Anger Out	13.9 (3.4)	13.8 (3.8)	.69
Anger In	15.0 (3.3)	15.4 (4.1)	.69
Anger Control	25.5 (5.3)	26.0 (4.8)	.71
Anger Expression	19.5 (9.4)	19.3 (9.4)	.93
Autonomic Perception	71.9 (32.3)	64.2 (31.7)	.09
Muscle Tension	32.1 (17.3)	25.2 (18.4)	.004
Toronto Alexithymia Scale	64.7 (12.0)	63.2 (12.4)	.47

**Table 2:** Demographic and medical characteristics, ETT parameters and psychological test scores by presence or absence of anginal symptoms during the positive exercise treadmill test.

	Type of ischemia during ETT		
Variable	Symptomatic	Silent	*P*
	(*N* = 90)	(*N* = 106)	
	%	%	
Male	87	87	.98
Caucasian	88	87	.84
History of Diabetes	13	16	.60
History of Myocardial Infarction	44	41	.58
History of Hypertension	53	42	.13
History of PTCA	31	39	.27

	M	M	
	(SD)	(SD)	

Age	62.5 (8.7)	62.5 (8.2)	.91
Total ETT Duration (mins)	6.9 (2.7)	8.4 (3.0)	<.001
Time to 1 mm ST Depression	4.8 (2.3)	5.9 (3.4)	.008
Time to End of Test from ST Onset	2.0 (1.5)	2.5 (2.6)	.03
Maximum St Depression (mm)	1.9 (0.6)	2.1 (0.6)	.04
Rate-Pressure Product at Onset of ST Depression (/1000)	21.8 (4.7)	23.7 (5.6)	.02
Rate-Pressure Product at Peak Exercise (/1000)	23.6 (4.5)	26.4 (5.7)	<.001
Peak Borg Exertion Score	16.1	16.2	.72
Peak SBP	177.8 (21.3)	184.6 (27.7)	.07
Peak-Resting SBP	36.6 (19.0)	42.2 (27.2)	.07
Beck Depression Inventory	7.3 (6.9)	4.7 (4.4)	.003
State Anxiety	33.9 (10.2)	29.5 (9.0)	.004
Trait Anxiety	34.0 (9.4)	31.3 (8.1)	.07
Modified Autonomic Perception	68.6 (32.6)	65.5 (31.8)	.49
Muscle Tension Perception	30.0 (18.9)	25.8 (17.6)	.09
Toronto Alexithymia Scale	64.7 (13.3)	63.0 (11.3)	.27
Anger Out	13.9 (3.4)	13.8 (3.7)	.79
Anger In	15.4 (3.8)	15.1 (3.9)	.56
Anger Control	25.5 (5.3)	26.1 (4.7)	.64
Anger Expression	19.9 (9.4)	18.9 (9.4)	.53
Cook-Medley Hostility Scale	21.0 (8.2)	19.2 (8.5)	.09
Harm Avoidance	12.8 (7.4)	11.0 (6.6)	.11
Novelty Seeking	16.1 (5.7)	16.9 (5.9)	.40
Reward Dependence	15.4 (4.1)	16.1 (3.8)	.18
